# Ageing gender-specific "Biomarkers of Homeostasis", to protect ourselves against the diseases of the old age

**DOI:** 10.1186/1742-4933-11-3

**Published:** 2014-02-06

**Authors:** Anna Maria Berghella, Ida Contasta, Giuseppe Marulli, Carlo D’Innocenzo, Ferdinando Garofalo, Francesca Gizzi, Marco Bartolomucci, Giacomo Laglia, Marisa Valeri, Mario Gizzi, Mauro Friscioni, Mario Barone, Tiziana Del Beato, Enzo Secinaro, Patrizia Pellegrini

**Affiliations:** 1 Istituto di Farmacologia Traslazionale (IFT), Consiglio Nazionale delle Ricerche (CNR), Unità Operativa di Supporto (UOS), via G. Carducci, 32 - Rotilio Center, 67100 L'Aquila, Italy; 2 Poliambulatorio “Casa della Salute” Nucleo San Gregorio, Azienda Sanitaria Locale (ASL) di Avezzano-Sulmona-L’Aquila, San Gregorio, AQ, Italy; 3 Facoltà di Medicina, Università Degli Studi G. D’Annunzio, Chieti-Pescara, Italy

**Keywords:** Age-related pathologies, Inflammation, Immunosenescence, Clinical homeostatic biomarkers, Gender immune pathways, Personalized therapies, Pathology risk indices, Prevention programs

## Abstract

Low-grade inflammatory state causes the development of the principal chronic-degenerative pathologies related with ageing. Consequently, it is required a better comprehension of the physiologic origins and the consequences of the low-grade inflammatory state for the identification of 1) the basic mechanisms that lead to the chronic inflammatory state and, after that, to the progression toward the pathologies and 2) the parallel identification of the prognostic biomarkers typical of these passages. These biomarkers could bring to several improvements in the health quality, allowing an early diagnosis and more effective treatments for: a) the prevention strategies on the healthy population, to assure a healthy longevity and b) the identification of personalized treatment in patients, to assure the benefit of the therapy. For the identification of these biomarkers it is necessary to consider that the ageing processes produce alterations of the physiologic systems and that these modifications compromise the communications between these networks: this state constitutes an obstacle for an appropriate physiologic homeostasis, that plays a fundamental role for the safeguard of the health. It is also to be considered that immune senescence affects both men and women, but it does it in different ways: a sexual dimorphism of immune pathways in the setting of immune response homeostasis is normally present, as we previously underlined. Therefore we hypothesize that, in order to prevent the development of the chronic-degenerative pathologies related with ageing, it is important to identify "Biomarkers of Homeostasis " specific for each gender: these are biologic molecules that should be measurable in a practical and no-invasive way and whose variations can quantify the male and female risk of losing the physiologic system homeostatic capacity. This competence is not only critical in the control of inflammation, but it is also prognostic for the passages from low-grade inflammatory state to the chronic inflammation and to the progression toward the degenerative pathologies. Beginning from the actual results, our intent is 1) to discuss and underline the importance of these new research perspectives in the definition of ageing gender-specific clinical "Biomarkers of Homeostasis" and 2) to propose homeostasis biomarkers, already present in the research results.

## Background

**Figure 1:**
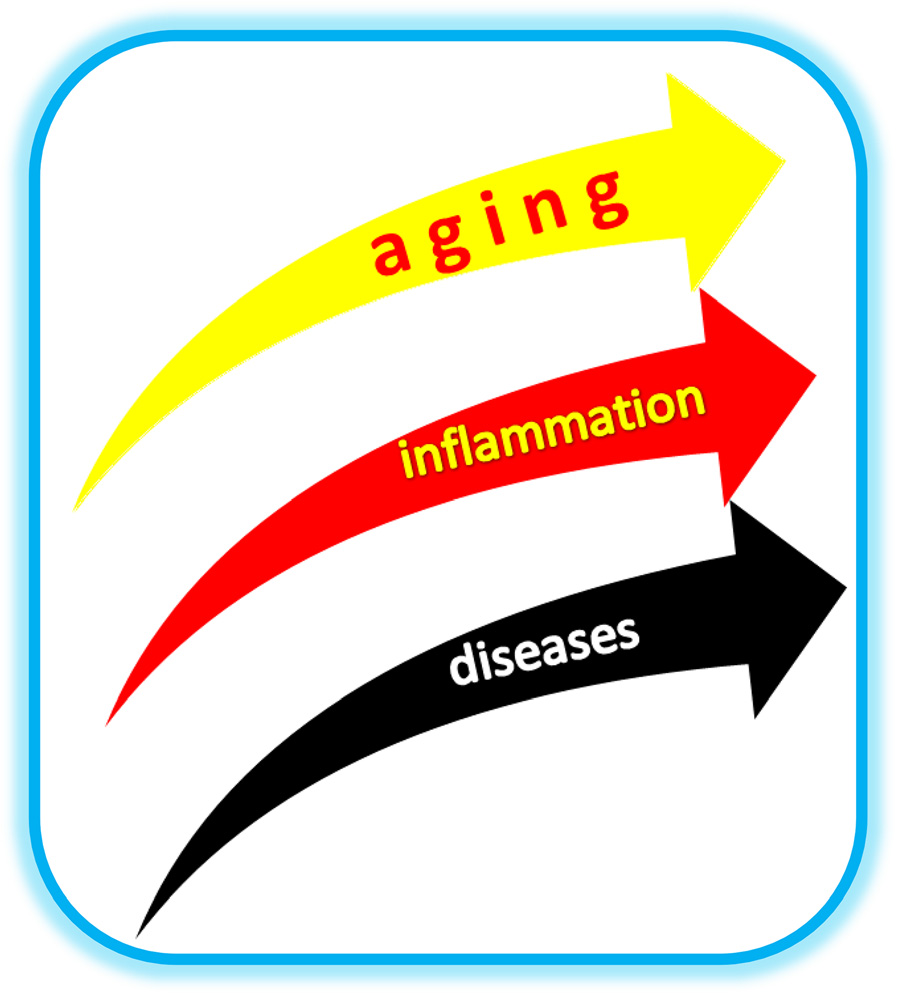
**Progressive increase of the elderly population: parallel increase of the subjects with chronic inflammation and chronic-degenerative diseases.** The mean age and the lifespan are progressively growing and this increase of the elderly population is related with a parallel increase of the subjects with chronic inflammation and chronic-degenerative diseases as neoplastic, autoimmune and neurodegenerative pathologies.

Mean age and lifespan are progressively growing and the increase of elderly population is related with a parallel rise of subjects with chronic inflammation and chronic-degenerative diseases (Figure 1) as neoplastic, autoimmune and neurodegenerative pathologies: it constitutes a challenge for the administration of the health system [1].

The difficulties in the administration of the chronic-degenerative diseases are related with the difficulties in the prevention and in the treatment of these pathologies: their diagnosis is arduous as the identification of a correct therapeutic line and, moreover, they absorb an increasing rate in the financial statements of the health assistance of the whole world, determining economic, social, biomedical and political problems [2].

**Figure 2:**
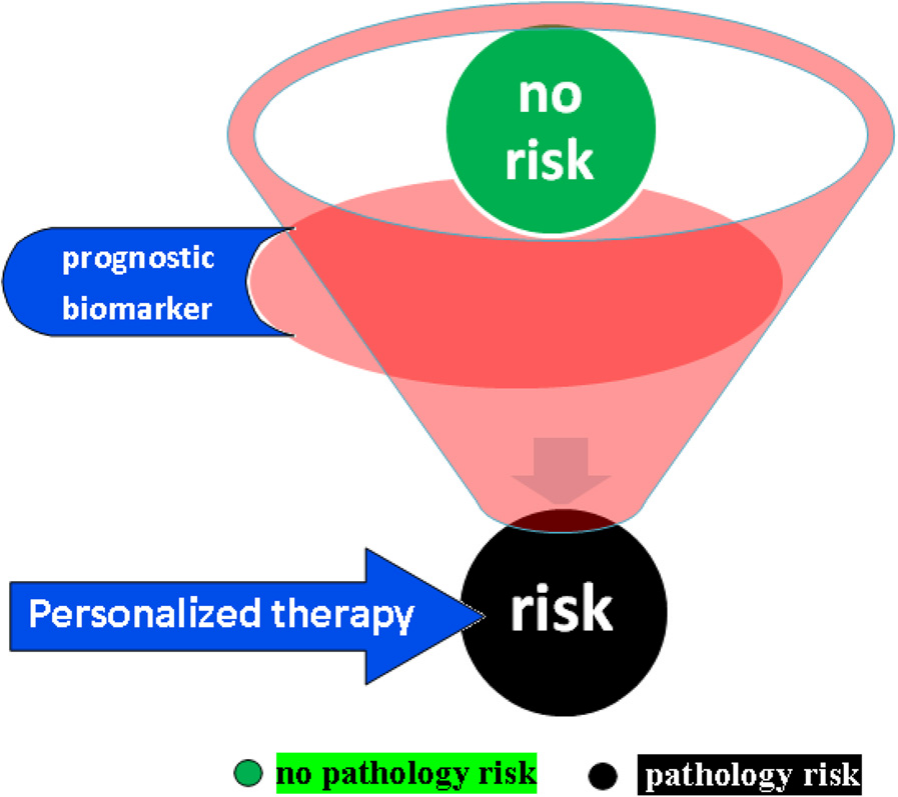
**Prognostic biomarkers could be suitable as risk indicator of pathologies and of the clinic/therapeutic trend.** The recent developments in the research, in particular in genetic, in proteomic and in informatics areas, are leading to the discovery of prognostic biomarkers that could be suitable as risk indicator of pathologies and of the clinic/therapeutic trend. The transport in the clinical practice of these biomarkers allows the early diagnosis and personalized therapeutic intervention, contributing, then, not only to the improvement in the health quality but also to a better administration of the sanitary system.

The recent developments in the research are leading to the discovery of prognostic biomarkers that could be suitable as risk indicator of pathologies and of the clinic/therapeutic trend (Figure 2). The transport in the clinical practice of these biomarkers allows an early diagnosis and personalized therapeutic interventions, contributing, then, not only to the improvement in the health quality, but also to a better administration of the sanitary system.

The prognostic biomarker is, then, a biologic highlighter that in clinical practice indicate 1) the risk of contracting a specific pathology, 2) the state of its progression and 3) the relative risk/benefit related with the specific therapy, exerting a central role in the selection of the most effective treatment. For these reasons, prognostic biomarkers have a significant role in clinical practice in which they are determining relevant progresses.

These progresses could be principally synthetized in the passage of the modern medicine from a generalized system, in which the therapy is the same for all the patients with the same disease, to a stratified organization, in which patients are subdivided in clinical/therapeutic subgroups, or, even better, to a personalized one, in which the treatment is defined on the specific physiologic characteristics of the single subject. This concept has relevant implication in clinical practice, thanks to the possibility of modern medicine, both stratified and personalized, to link the survey of a specific prognostic biomarker to a specific pathology and to its related therapeutic risk/benefit ratio (Figure 2).

For this success, however, it is necessary to define suitable prognostic biomarkers, with a real disease predictive power that could be used to better define translational protocols in the routine clinical practice. They have to be detectable in a no-invasive and early way in relationship with the output. It is clear at this point that the definition of these biomarkers constitutes a clinical and social necessity and it is an important competence of the scientific research in this area.

### Presentation of the hypothesis

The definition of these biomarkers is currently possible, thanks to new technologies that allow us to reveal the complexity of the biology of our physiologic (the normal health state) and pathologic (disease states) systems: in this way it is possible to select the biomarkers that are significant for the clinical monitoring of the two states, physiologic and pathologic.

Being an appropriate physiologic homeostasis essential for the health safeguard, we can define these clinical biomarkers thanks to the above mentioned procedures and studying the homeostatic state: 1) the homeostatic balance of the biologic system in the healthy state (the biomarkers of the physiologic state) and 2) the loss of this homeostasis in the pathological ones (the biomarkers of the pathological degeneration and of its progression).

### Research perspectives for the definition of clinical "Biomarkers of Homeostasis”: for early diagnosis and more effective treatments in the ageing related chronic-degenerative diseases

**Figure 3:**
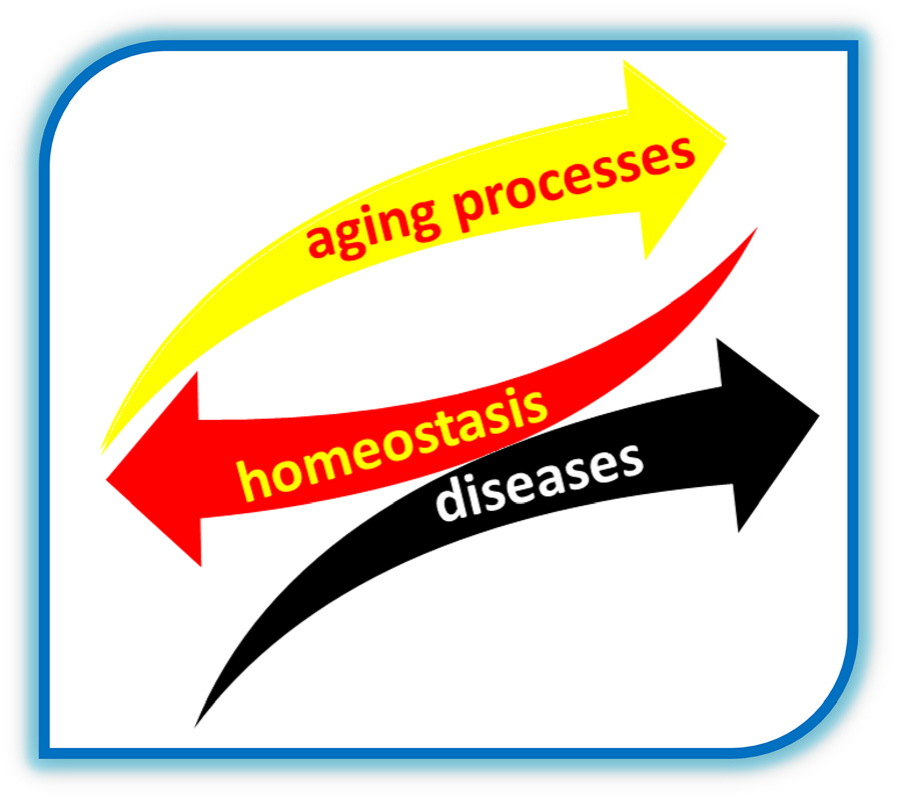
**Ageing research data identify the incapacity to preserve health in the loss of homeostasis.** Changes of the physiologic functions related with the ageing process obstacle an appropriate physiologic homeostasis, impeding health safeguard: they affect all the cells and in particular the nervous, endocrine, immune ones, compromising the functioning of these fundamental regulatory system and their mutual communication.

There are two essential points that give support to these new research perspectives:

1) Changes of the physiologic functions related with the ageing process obstacle an appropriate physiologic homeostasis (Figure 3), impeding in this way the health safeguard: they affect all the cells and in particular the nervous, endocrine, immune ones, compromising the functioning of these fundamental regulatory systems and their mutual communication [3-6].

2) Numerous studies are underlining a strong relationship between the inflammatory mediators and the ageing-related diseases [1] (Figure 1), as cardiovascular pathologies and Alzheimer, and they show that genetically predisposed individuals that are able to control inflammatory activity have a reduced possibility to develop inflammatory ageing-related diseases, having a greater chance to become centenary [7-11].

Inflammation is a complex process that involves widespread changes in cellular and molecular components of physiology. Although controlled inflammation is a necessary process that is required for an array of processes including tissue repair, wound healing and for defence against invading foreign pathogens, chronic and uncontrolled inflammation is harmful and has now been linked to a number of human ailments [12,13] (Figure 1).

Different studies show that a “low grade systemic inflammation” characterizes ageing [1] and that inflammatory biomarkers are significant prognostic indices of mortality in elderly subjects [1,14-16]: The low grade systemic inflammation is involved in different biologic mechanisms that are responsible for the decline of the physical function and for the age related pathologies as Alzheimer or atherosclerosis that origin and progress thanks to this type of inflammatory state [1,7,8,16-18]. A large class of factor, as smoke, infections, obesity, genetic and the decrease of the sexual hormones could contribute to the systemic low-grade pro-inflammatory state, typical of elderly individuals [15]. The increase of the circulating inflammatory mediators levels could also derive from a constant low-grade activation of the immune system caused by a chronic exposition to different types of pathogens [19,20]. Some studies linked the exposition to infections both the inflammatory state and the increased risk of cardiac attack, ictus and cancer [21-24].

Consequently, research data indicate that during ageing 1) the incapacity to preserve health, is caused by the loss of physiologic homeostasis and that 2) the base for the origin and the progression of chronic-degenerative pathologies is the chronic inflammation (Figure 1).

### Implication of the hypothesis

As a consequences, it is in the physio-pathological pathways that lead to chronic inflammation that it is to be researched the scientific ratio for the definition of clinical biomarkers of homeostasis, for the prevention and the treatment of chronic-degenerative diseases related with ageing.

**Figure 4:**
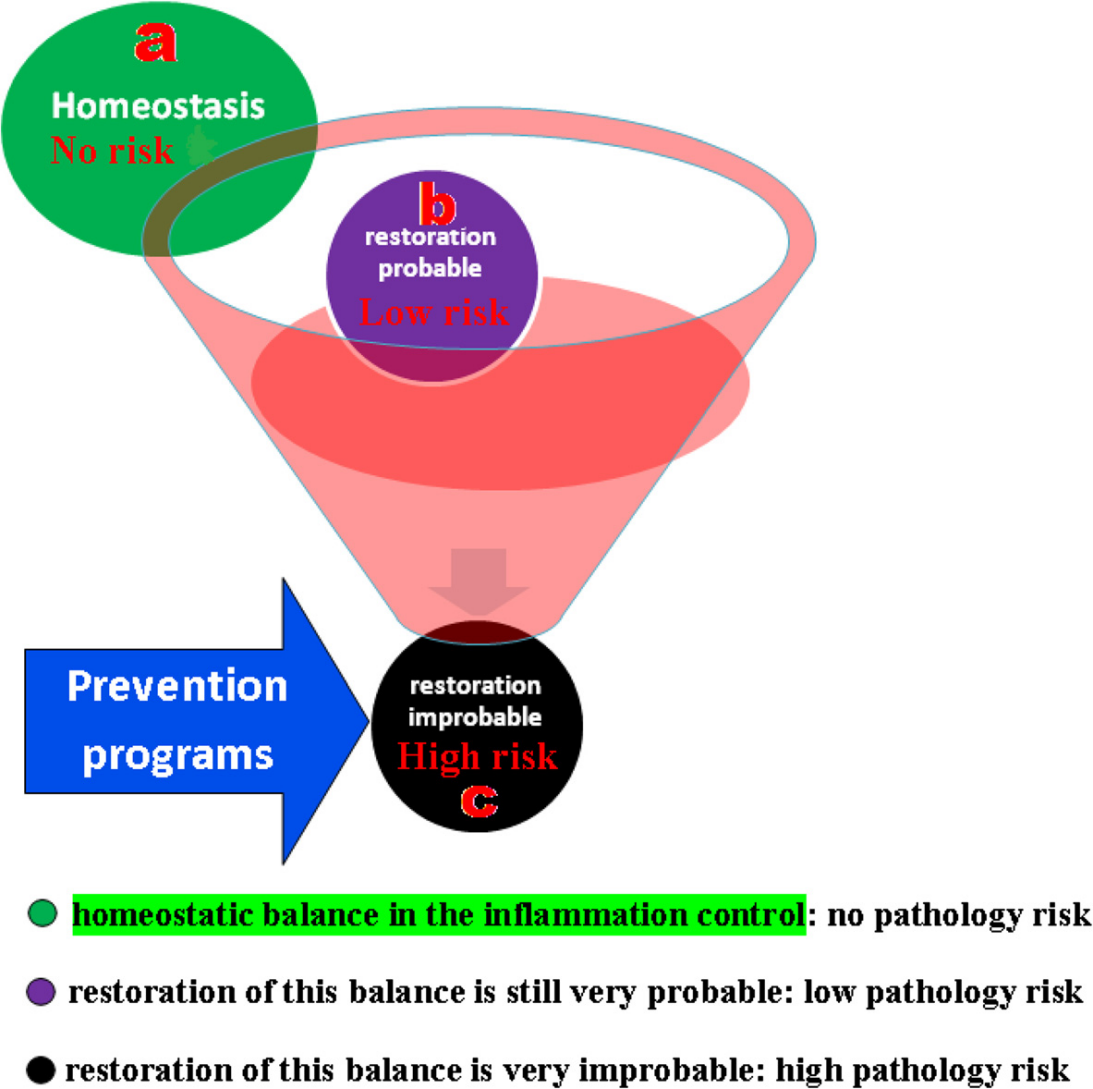
**It is in the physio-pathological pathways that lead to chronic inflammation that it is to be researched the scientific ratio for the definition of clinical biomarkers of homeostasis for the prevention and the treatment of chronic-degenerative diseases.** The procedure consists in the individuation of biologic molecules, whose variations could quantify the risk of losing the physiologic system homeostatic capacity in the control of the inflammation. These molecules have to be measurable in a practical and no-invasive way, in all the following stages: **a)** in the normal state of health, in which we identify biomarkers of a type that are in this case indices of no-risk of disease; **b)** in the transient inflammatory state, where we define biomarkers of b type that are indices of low-risk of pathology; **c)** in the chronic inflammatory state, in which it is possible to select biomarkers of c type that are indices of high-risk of disease. The scientific rational is that prognostic biomarkers have to be predictive for the following passages to be efficient in the prevention of chronic-degenerative diseases: 1) from the health physiologic condition (typical for the a type biomarkers), in which there is a homeostatic balance in the inflammation control, 2) to the transient inflammation (b type) where the restoration of this balance is still very probable, 3) and/or to the chronic inflammation (c type) in which this recovery is physiologically very improbable, while it is high the risk of progression toward degenerative pathologies.

More specifically, using the methodologies of the study of the system biology, the procedure consists in the individuation of biologic molecules, whose variations could quantify the risk of losing the physiologic system homeostatic capacity in the control of the inflammation (Figure 4). These molecules have to be measurable in a practical and no-invasive way, in all the following stages: a) the normal state of health, that should be monitored by biomarkers that indicate no-risk of pathology and that we define of a type; b) the transient inflammatory state, indicated by biomarkers linked to low-risk of pathology, that we consider of b type; c) the chronic inflammatory state, defined by biomarkers indicating a high-risk of pathology, considered of c type. The scientific rational is that, to be efficient in the prevention of chronic-degenerative diseases, prognostic biomarkers have to be predictive for the following passages: 1) from the health physiologic condition, in which there is a homeostatic balance in the inflammation control (typical of the a type biomarkers), 2) to the transient inflammatory state, where the restoration of this balance is still very probable (b type biomarkers), 3) and/or to the chronic inflammatory state, in which this recovery is physiologically very improbable (c type biomarkers), while it is relevant the risk of progression toward degenerative pathologies (Figure 4).

**Figure 5:**
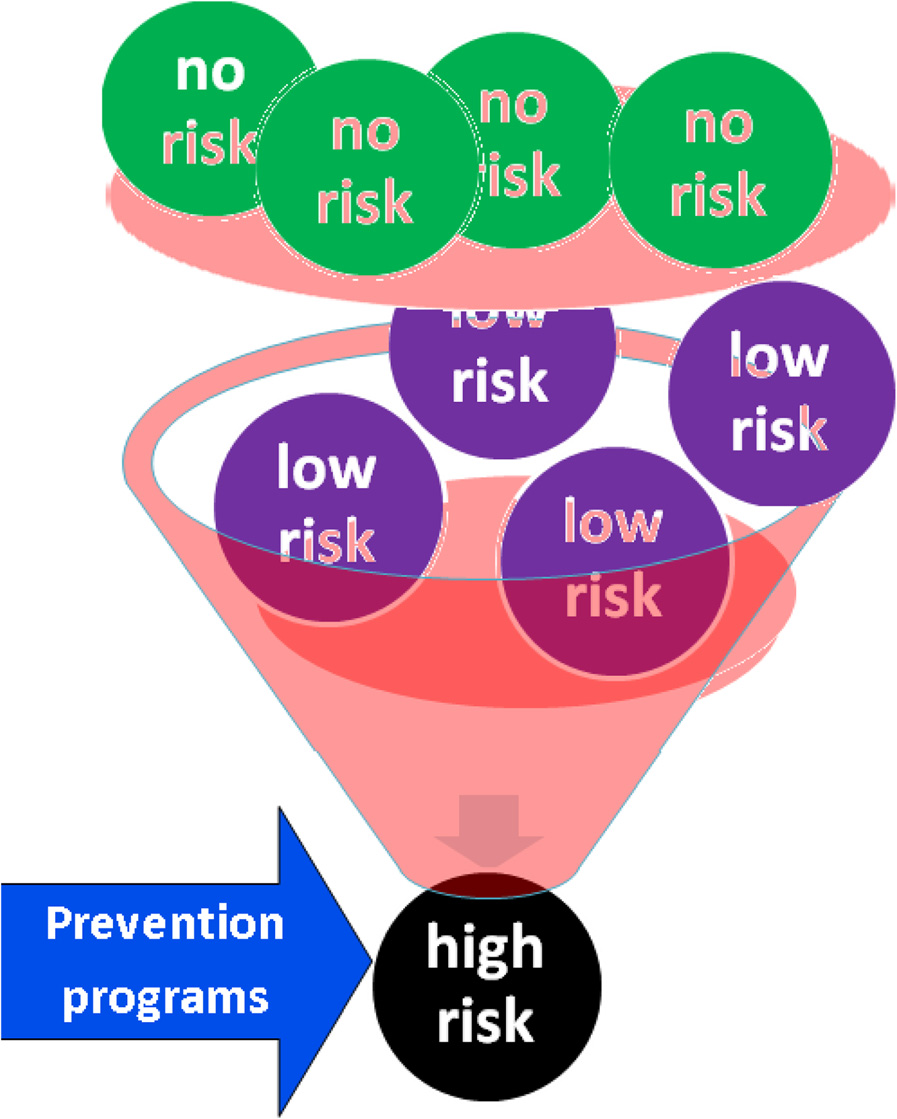
**Biomarkers of homeostasis allow an early diagnosis and personalized therapeutic interventions, permitting a promising change in the clinical practice and in the sanitary system administration.** They open to a large class of prevention programs for these pathologies on the healthy population, that are not yet available, because they are suitable for the identification of subjects that are still healthy, but with a high risk to develop this kind of pathologies. On these individuals it is justified the application of preventive sanitary procedures, saving the actuation where they are not motivated.

These biomarkers allow an early diagnosis and personalized therapeutic interventions, permitting a promising change in the clinical practice and in the sanitary system administration. They open to a large class of prevention programs for these pathologies on the healthy population, that are not yet available, thanks to their usefulness in the identification of subjects that are still healthy, but they are considered in risk of developing chronic pathologies (Figure 5). On these individuals it is, in fact, justified the application of preventive sanitary procedures, saving the actuation where they are not motivated. Moreover they are prognostic for the patient stratification in clinic/therapeutic subgroups, because they also permit the quantification of the risk/benefit related to a therapeutic treatment, allowing the development of the personalized medicine that could lead to a positive transformation of the clinical strategies: the risk or benefit of the therapy is connectable to the inability or ability of the specific treatment to restore physiological homeostasis, that is underlined by the changes of the individual biomarker from b or c types to a type.

These aims could be reached identifying the molecules that have the function of homeostatic biomarkers in the control of the inflammation by evaluating two principal groups, subdivided in internally homogeneous layers for gender and age class: 1) the group of healthy subjects and 2) the group of patients affected by chronic degenerative pathologies. In fact, the biomarkers of a healthy longevity have to be identified on healthy individuals, but the biomarkers of the degeneration toward the pathology and of its progression have to be recognized on patients, being the chronic-degenerative pathology a pathological development of the basal inflammatory process that begin their activity when the subject is still healthy [25,26].

**Figure 6:**
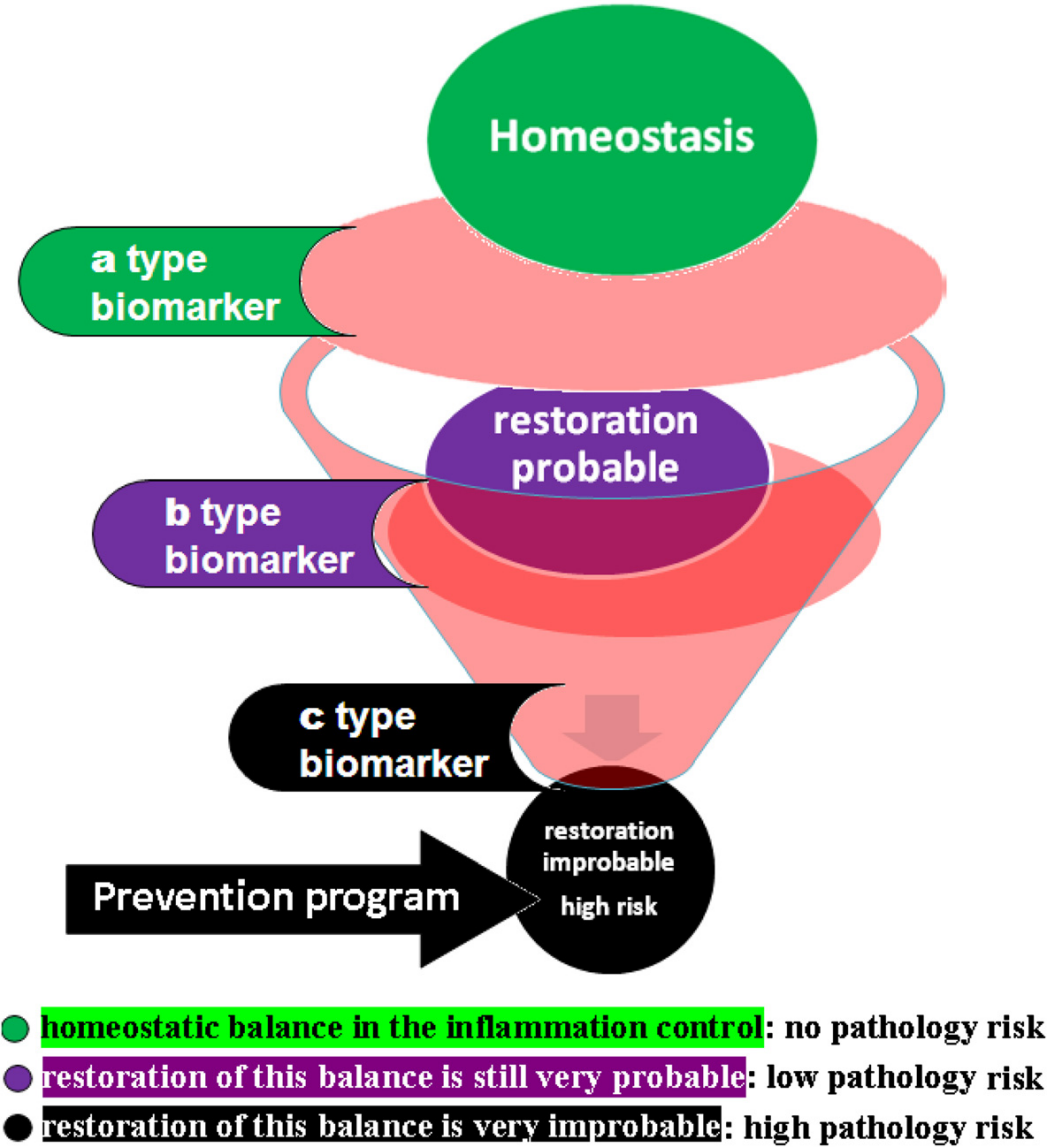
**The identification of molecules that have the function of homeostatic biomarkers.** The study of the relationships between ageing and a healthy longevity, in the healthy population comparing the age subgroups, could be useful in the definition of 1) a type biomarkers (prognostic for the homeostatic state and of no-risk of pathology) and 2) b type biomarkers (prognostic for the transient inflammatory state, then of the restoration possibility of the homeostatic condition and of low-risk of pathology). These biomarkers are usable in prevention intervention on the healthy population and in the evaluation of the risk/benefit for the therapeutic selection of patients. On patients with chronic-degenerative pathologies the procedure is to select the biologic molecules with homeostatic biomarker capacity in association with the losing of the inflammation control, comparing the patient groups and the healthy individuals in internally homogeneous layers for gender and age, as upper described. The intent is to define the significant molecules to select the c type of homeostatic biomarkers, indices of high-risk of disease, prognostic for the degeneration toward pathology.

As a consequence, for the identification of these molecules could be valid the following procedure: 1) To select the biologic molecules with homeostatic biomarker capacity in the control of inflammation, we need to study the relationships between ageing and a healthy longevity in the healthy population, comparing the age subgroups as follow: the age subgroup in which the physiologic system is healthy and where it has the optimal homeostatic functioning (for example 18–55), with 1) the age subgroup in which the physiologic system is still healthy, but its functional capacity is negatively influenced by the ageing effects (65–85), and also with 2) the age subgroup in which the physiologic system is still healthy, but the homeostatic capacity in the inflammation control is able to stem the ageing effects and the age related pathologies (>95). The aim is to discover the significant molecules for this success: they are associated with better control of inflammation and they allow to escape major age related diseases. This could be useful in the definition of 1) the a type biomarkers (prognostic for the homeostatic state and no-risk of pathology) and 2) b type biomarkers (prognostic for the transient inflammatory state, but also for the restoration possibility of the homeostatic condition and, then, for low-risk of pathology) (Figure 6). These biomarkers are usable in prevention intervention on the healthy population, to select subject with low-risk of pathology and in the evaluation of the relative risk/benefit related with the specific therapy, in the selection of the most effective treatment: the success or not of the treatment is connectable to the ability or inability to restore physiological homeostasis, underlined by the ability or inability to determine a change of the homeostasis biomarkers in the examined subject, from the b to the a type.

The subgroup of subjects older than 95 shows physiologic characteristics that support our hypothesis: it has been demonstrated, in fact, that centenarians have better chances to escape major age related diseases, including cancer [27]. Autoptic studies clearly showed that in centenarians the cause of death by chronic-degenerative diseases was lower than expected [28] and was decreasing in individuals over 99 years, compared to younger ones [29]. Therefore, advancing age is known as the most potent inductor of chronic disorders [30], but in old individuals this prevalence does not increase, despite the longer exposure to exogenous and endogenous factors that could be able to cause pathologies. Centenarians are characterized by a higher frequency of markers associated with better control of inflammation [31]: the reduced capacity of centenarians to mount inflammatory responses appears to exert a protective effect towards the development of those age-related pathologies having a strong inflammatory pathogenetic component. Centenarians seem to carry a genetic background with a peculiar resistance to cancer which is also an anti-inflammatory profile [31].

2) In order to select biologic molecules that can be used as biomarkers in the loss of homeostasis and of the inflammation control, we need to study the relationships between ageing and the disease onset and progression: it can be examined comparing patient groups and the healthy individuals in internally homogeneous layers for gender and age, as upper described, and for disease stage. The intent is to define the significant molecules to select the c type of homeostatic biomarkers, indices of high-risk of disease, prognostic for the degeneration of transient inflammatory state toward chronic inflammatory state and the degenerative pathologies (Figure 6).

These biomarkers are usable in prevention intervention on the healthy population, in order to select subject with high-risk of pathology and to individuate the most effective treatment for the patient: the success or not of the therapy is connectable to the ability or inability to restore physiological homeostasis, then to the ability or inability of the treatment to determine a change in the homeostasis biomarkers in the examined individual from c to an a type, considering a change toward the b type an indication of a not idoneus therapy or not sufficient dosages.

### For the definition of clinical "Biomarkers of Homeostasis" the evaluation has to be necessarily made separately in men and women

**Figure 7:**
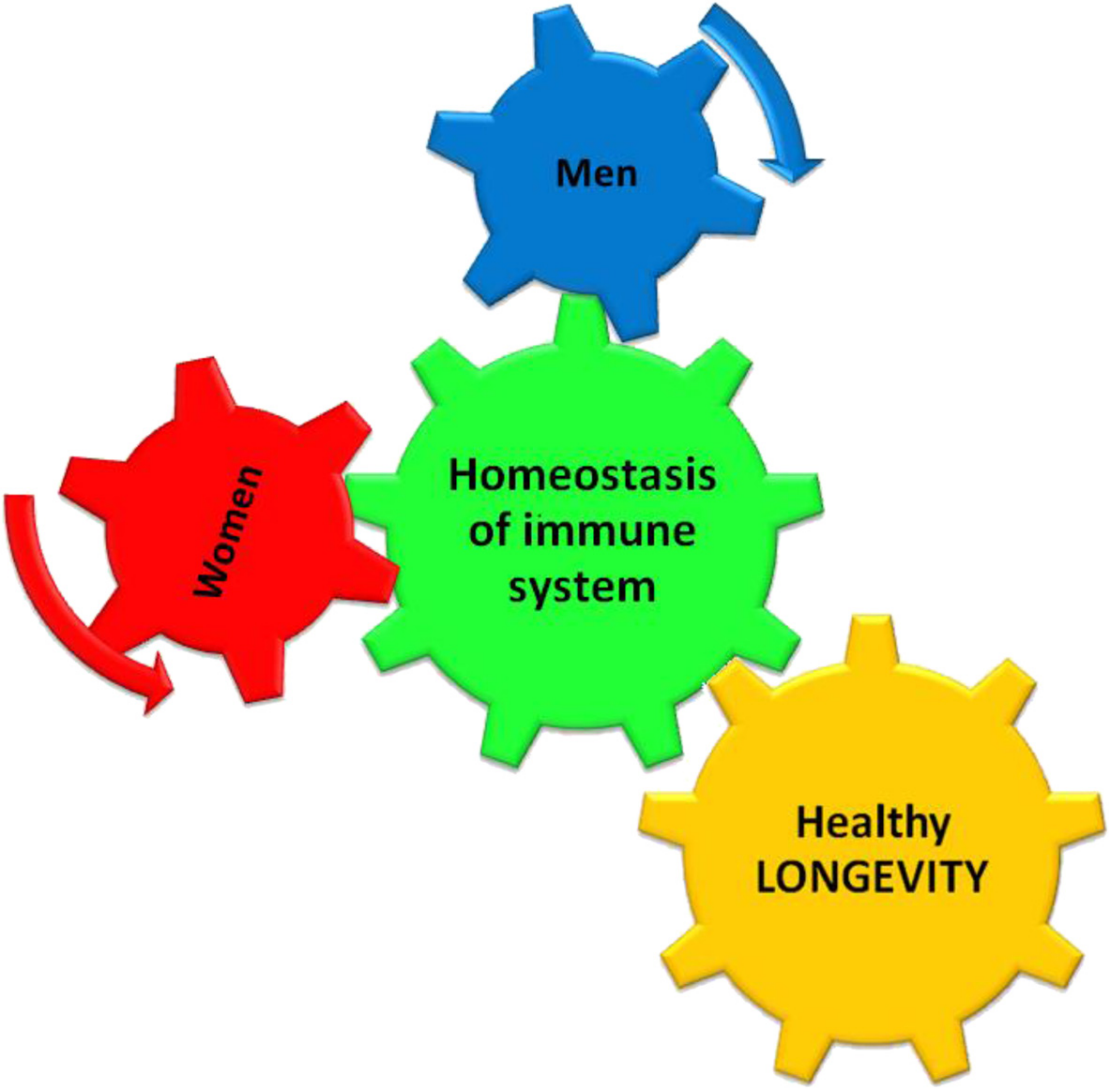
**The Importance of the evaluation of the both gender, men and women, as independent groups: men and women follow different strategies to reach longevity.** Clinical and experimental data show that, when the immune system is involved, men and women could not be evaluated in only one group, because the results would be not real, since in the immune response there is a natural gender dimorphism. It has been proved that the immunosenescence affects both men and women, but it afflicts them differently. Furthermore, it has been demonstrated that female and male hormones act affecting in opposite ways the immune system. The research has shown that the gender, male or female, is associated with relevant incidence and prevalence of different types of age related diseases and it also is an important variable in the genetic of longevity, indicating an important observation: men and women follow different strategies to reach longevity.

In the comprehension of the relationships between ageing and 1) the healthy longevity and 2) the chronic-degenerative diseases, it is important to consider that a healthy longevity is related with a correct functioning of the immune system [32-35] and that the pathology is generated by alterations of this one. In this contest it is also to be considered that it is now assured the existence of a gender dimorphism in the homeostatic control of the immune system: men and women not only follow different physiological pathways for the regulation of the immune response, but these pathways, specific for each gender, also suffer alterations during ageing and they predispose differently men and women to diseases [34] (Figure 7).

Clinical and experimental data show the presence of a gender dimorphism in the immune response [36-39]: male and female hormones influence the immune system and they behave in opposite ways [40-42] (Figure 7). The research has revealed that the gender is associated with important incidence differences and with a prevalence of differential age-related pathologies [43]. The sexual dimorphism is also a fundamental variable in the genetic of longevity [44-46], indicating that men and women follow different strategies for a healthy longevity success (Figure 7).

For these reasons, in the selection of significant molecules for the definition of clinical biomarkers of homeostasis, the evaluation has to be necessary made separately in men and women, as different independent groups.

### For the study of the immune system homeostasis it is relevant the cytokine survey

For the study of the immune system homeostasis it is relevant the cytokine survey, being these substances produced by cells of this system and regulating the transposition of the information between cells, thanks to the activation of membrane receptors.

There is a growing consensus that the big part of chronic pathologies of ageing are linked by a common biology and that cellular senescence and the related secretion of inflammatory mediators are common factors [1,17,18]. It has become increasingly apparent that the cellular senescence is a cellular stress response with a complex phenotype and the senescence-associated secretory phenotype entails the secretion of numerous proteases, growth factors and cytokines which can have beneficial or detrimental effects, depending on the physiological context [1,17,18].

Moreover, recent studies [47-49] indicate, for the first time, that the homeostatic equilibrium of the immune response is regulated by cytokines that differ in men and women, and that it is attributable to these differences the different gender trend in the 1) immune response, 2) the predisposition to diseases and 3) the therapeutic response, opening to a new area for the translational research at this level.

In fact these data show that the IFNy cytokine regulates the male immune system homeostasis, while the IL6 cytokine regulates the female one [47-49].

**Figure 8:**
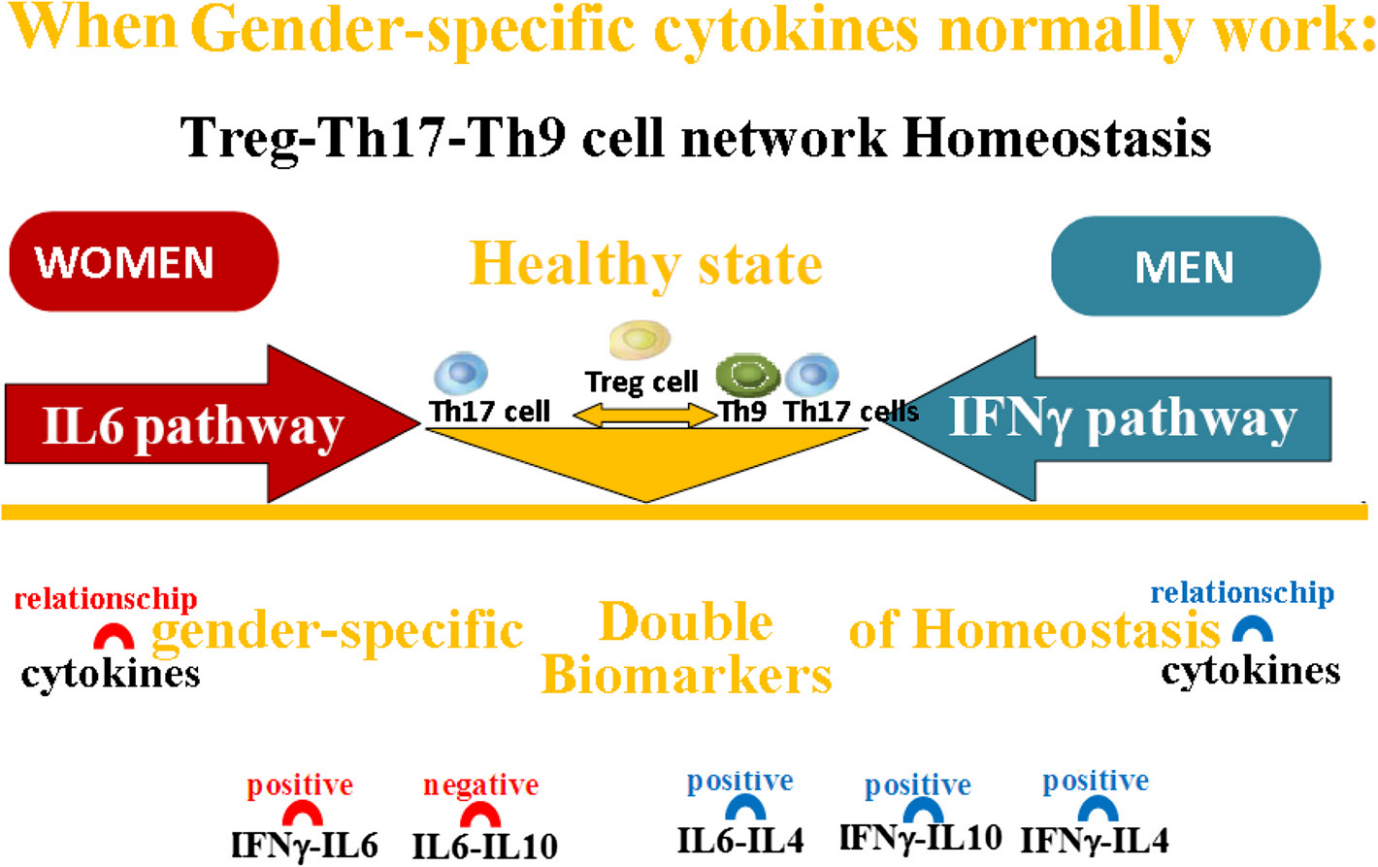
**Homeostasis is preserved and there are no differences between men and women in the outcome of the immune response when the pathways of the gender specific cytokines (IFNγ and IL6) still normally work.** The relevance of the gender specific differences in the regulation of the immune response is underlined by the evidence that homeostasis is preserved and there are no differences between men and women in the outcome of this response when the pathways of the gender specific cytokines (IFNγ and IL6) still normally work. It has been shown that the variations between pro- and anti-inflammatory cytokines could influence the success of the immune response. The most relevant discover, however, for the definition of suitable prognostic biomarkers, is the identification of “double prognostic biomarkers”: they are constituted by couple of pro- and anti- inflammatory cytokines that differ between men and women and assure the success of the immune response varying in appropriate relationship each other and following different pathways in each gender.

The above mentioned study [47] explains that a different gender susceptibility and clinical course in diseases is caused by different polarization of Th cell subsets (Treg, Th17 and Th9) (Figure 8), determined by the interactions of TGFβ, IL6, IFNy, IL10 and IL4 cytokine pathways which vary between men and women [47]. These findings are supported by the studies, demonstrating that there is a reciprocal development relationship between Treg, Th17 and Th9 cell subsets, for the reason that: i) TGFβ triggers the expression of Foxp3 transcription factor in naïve T cells, generating Treg cells, but ii) IL6 inhibits the TGFβ driven expression of Foxp3; TGFβ together with IL6 induce ROR-gt transcription factor, triggering the developmental program of Th17 cells [46]; ii) IL4 also inhibits TGFβ induction of Foxp3 expression, but TGFβ together with IL4 induce the differentiation of Th9 cells, which produce IL9 cytokine. Autoimmune disease susceptibility in women, such as multiple sclerosis, has been related with the influence of IL6 which plays a key role in autoimmune diseases, since it is a T cell differentiation switch factor for Tregs and Th17 cells [48-51]. The greater probability in men of developing the primary progressive multiple sclerosis form [52], on the other hand, has been associated to the influence of IFNy on Th9 cell inhibition: co-expression of IL-9 and IL-17 was identified as a novel Th17 function in mediating autoimmune tissue destruction [53,54]. Recent research on multiple sclerosis disease [55] confirms these data, showing that a sexual dimorphism in autoimmune diseases is the result of different cytokine pathways that regulate the Th cell network homeostasis. Indeed, these results indicate that: IL6 pathways are involved in Treg cell imbalance and produce a worsening of the neurological deficit in both men and women groups of multiple sclerosis patients, but the efficacy of IFNy-treatment in the re-establishment of The cell network balance (the immune response homeostasis) and delaying the progression of neurological disability (the neurological response homeostasis) is linked to the re-establishment of IL6 pathway in women and of IFNγ pathway in men [55].

**Figure 9:**
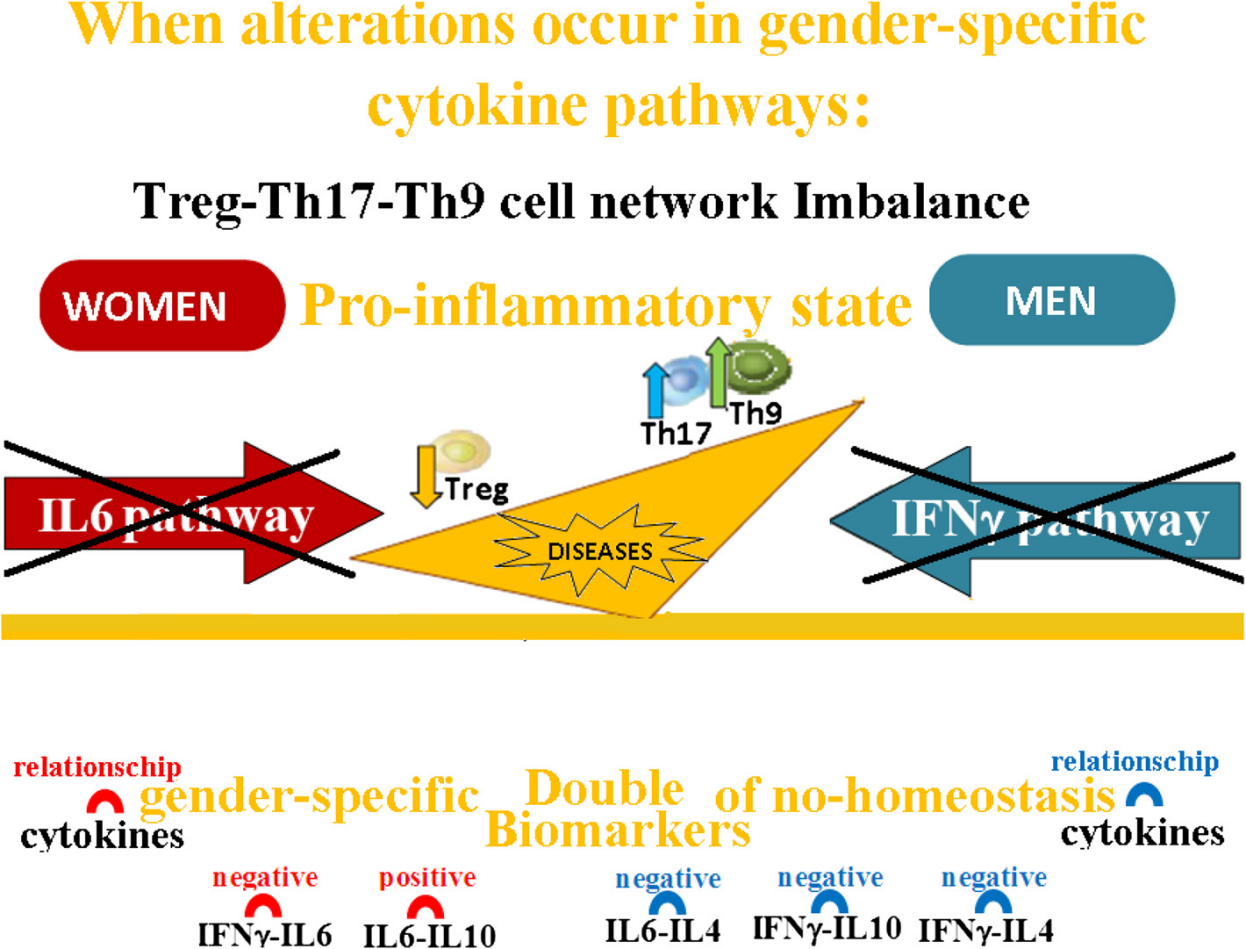
**When alterations occur in the pathways of the gender-specific cytokines, the consequences for men and women are different, in terms of disease development.** This event is related to the impairment of the immune system homeostasis, because the alterations of gender-specific cytokine pathways cause a pathologic polarization of specific T cell types, different for each gender. The reason of this fact is related to the different effects generated by IFNγ and IL6 cytokines, that are present in the cellular environment, on the generation of Th cell subtypes during the immune response.

The correct functioning of the IFNy pathway in men and IL6 pathway in women is the homeostatic biomarker for the ascertainment of the immune system homeostasis in the control of inflammatory state and, as a consequence, it is also related to a healthy longevity (Figure 8). It has been found that an altered state of these parameters (Figure 9) could be a prognostic biomarker for the passage from the healthy condition to the onset of an adenoma and it could be an index of the progression toward colorectal cancer [47], and for an aggravation of the neurological deficit in multiple sclerosis patients [55]. On the other side, the good functioning of the IL10 cytokine regulate the restoration of the immune system to the rest state homeostasis, after the response, both in men and women. IL10 could be a prognostic biomarker of a healthy longevity both in men and women, but only if IFNy and IL6 are correctly functioning in the respective gender: if this condition is not respected IL10 is a biomarker for the tumor progression in both genders [47,55].

### Ageing gender-specific clinical "Biomarkers of Homeostasis" that could be used in interventional studies aimed at improving health span

#### Inflammatory—anti-inflammatory cytokines serum levels

An applicable discovery for the definition of the upper described clinical biomarkers of homeostasis has been the identification of “double homeostatic biomarkers”, defined as pro and anti-inflammatory cytokine couples, specific for each sex, that control the immune system homeostasis and the inflammatory process in the respective gender by varying in an appropriate way [47,55-58].

**Figure 10:**
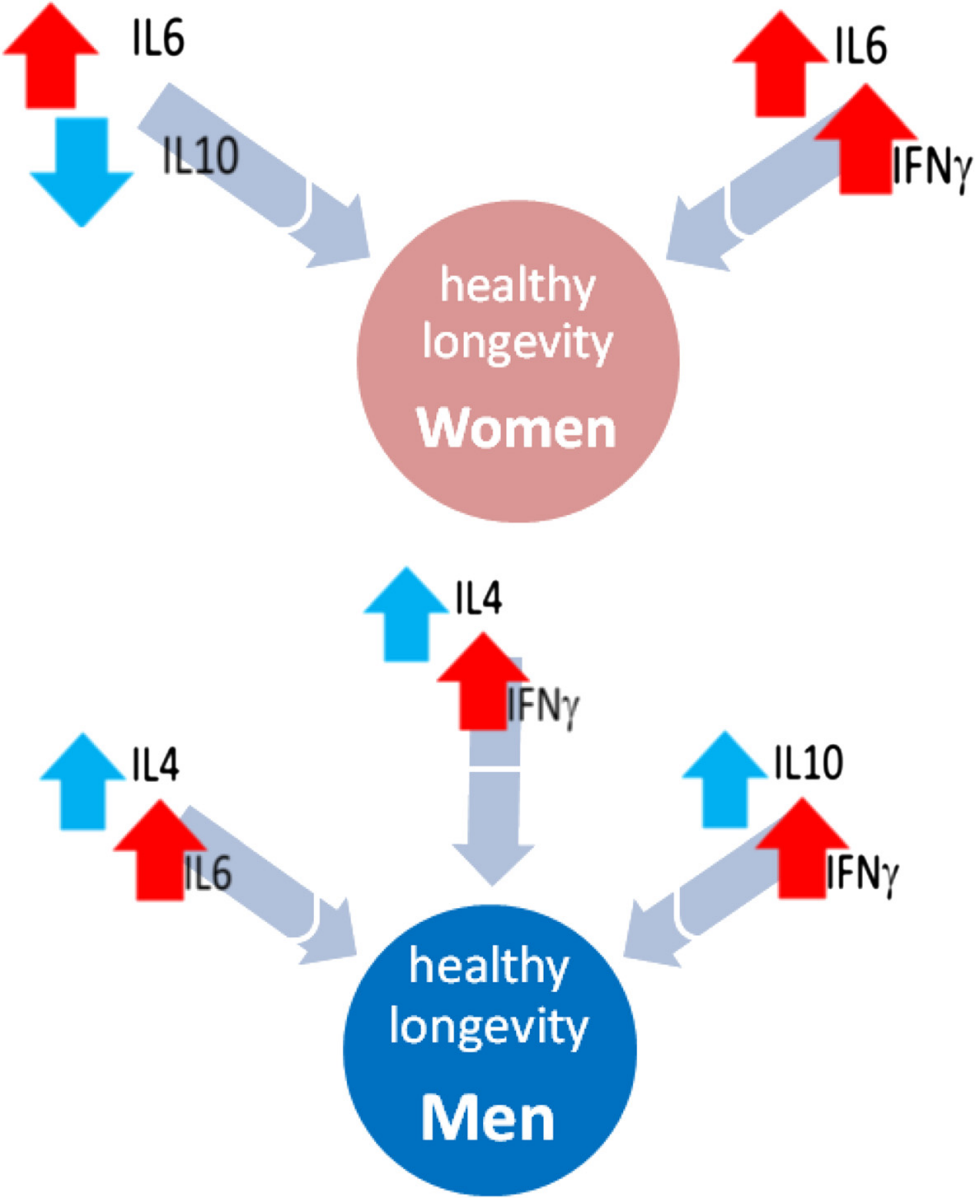
**The variations between pro- and anti-inflammatory cytokines could influence the success of the immune response.** The most relevant discover, however, for the definition of suitable prognostic biomarkers, is the identification of “double prognostic biomarkers”: they are constituted by couple of pro- and anti- inflammatory cytokines that differ between men and women and assure the success of the immune response varying in appropriate relationship each other and following different pathways in each gender. The early evolution of the immune response is influenced by the positive correlation between the production of IFNγ-IL10 and IL6-IL4 in men, and the negative one between IL6-IL10 in women. The evolution of the late response is also influenced by the positive correlation between the production of IFNγ-IL4 in men and IL6-IFNγ in women. More specifically the “double prognostic biomarkers” for men are related by direct proportionality (they both increase or decrease together with the same trend, both positive or negative) between the IFNγ―IL10, IL6―IL4 and IFNγ―IL4 cytokine levels; while they are interconnected in women by an inverse proportionality (an increase of the first correspond to a decrease of the second and vice versa) between the IL6―IL10 cytokine levels, and with a direct proportionality between the IL6―IFNγ cytokines. These variations between the couple of inflammatory and anti-inflammatory cytokines, specific for each gender, are to be considered “double biomarkers” gender-specific, in the valuation of relationships between ageing and a healthy longevity.

More specifically [52,55,56], in male peripheral blood these variation are defined with a direct proportionality (they both increase or decrease with the same trend, positive or negative) that correlates the levels of **IFNγ―IL10, IL6―IL4** and **IFNγ―IL4 cytokine couples** (Figures 9 and 10); while in women these variations in the peripheral blood interest the levels of **IL6—IL10** couple with a relationship of inverse proportionality (the increase of the first relates with the decrease of the second and vice versa), while there is a direct proportionality between **IL6—IFNy couple** (Figures 8, 9 and 10).

### The thioredoxin 1 (Trx1) and the soluble molecule of CD30 receptor (sCD30)

The study of the relationships between the redox state and the functioning of the immune cells and the individual longevity, has a significant role for the comprehension of the ageing process finalized to a healthy ageing [57-61]: one of the cause responsible for the differential gender susceptibility to pathologies is the different capacity of male and female cells to defend themselves toward the oxidative stress [62,63].

These results show that the cells of both sexes are very different in terms of reactive oxygen species (ROS) production and of their susceptibility to the oxidative stress and it could constitute a new promising area of research. Oxygen metabolism could lead to the production of ROS in each type of cells, also in the immune system ones: in fact they present antioxidant compounds and enzymes (as glutathione and thioredoxin reductase) [64,65], that are able to neutralize ROS and preserve the oxidative cellular balance. However, the activity of ROS seems to be regulated differently in men and women and it could be directly influenced by sexual hormones [63].

These results indicate, then, that the homeostatic biomarkers are selectable in the peripheral blood factors constituting a correlation between the redox and the immune system and that are involved in multiple cellular processes, as proliferation, cellular cycle and the pathways of death and survival signals [66,67].

The Thioredoxin system (Trx) is a fundamental physiologic regulator of the redox factors for the immune response [67-69]. Trx1, a protein containing selenocisteine, catalyzes the NADPH-dependent reduction of Trx1 reductase (TRrx1) and several other oxidized cellular proteins [70-72]. After an oxidative-stress, Trx1 gives origin to cellular signals, activating specific transcriptional factors that regulate the decode in the nucleus of the genes that produce substances for the defense of the cell against the ROS, that have induced the oxidative stress condition [71-74].

As a consequence, in order to select the clinical homeostatic biomarkers related with the inflammation control, it is clinically relevant to underline that the CD30 receptor (RCD30 on the immune cell membrane (T and B cells, monocytes, dendritic cells, NK, eosinophils and granulocytes), is a specific Trx1 receptor [75].

There are indications that the physiological function of RCD30 could behave as a signal transducing molecule. The interaction between RCD30 and its ligand (CD30L) on the T and B activated cell, monocytes, neutrophils, eosinophils induces the rapid activation of genetic transcriptional factors as JunN-kinase and NF-kB [76,77]. It has been shown that RCD30 signals induce and regulate the integrated lymphocytary genetic expression of molecules that have a cytotoxic effect, they control lymphonodal traffic, proliferation and apoptosis [78]. RCD30 is a member of the TNF/NGF receptor super-family, it is generally defined as a molecule that mediates the regulation signals.

**Figure 11:**
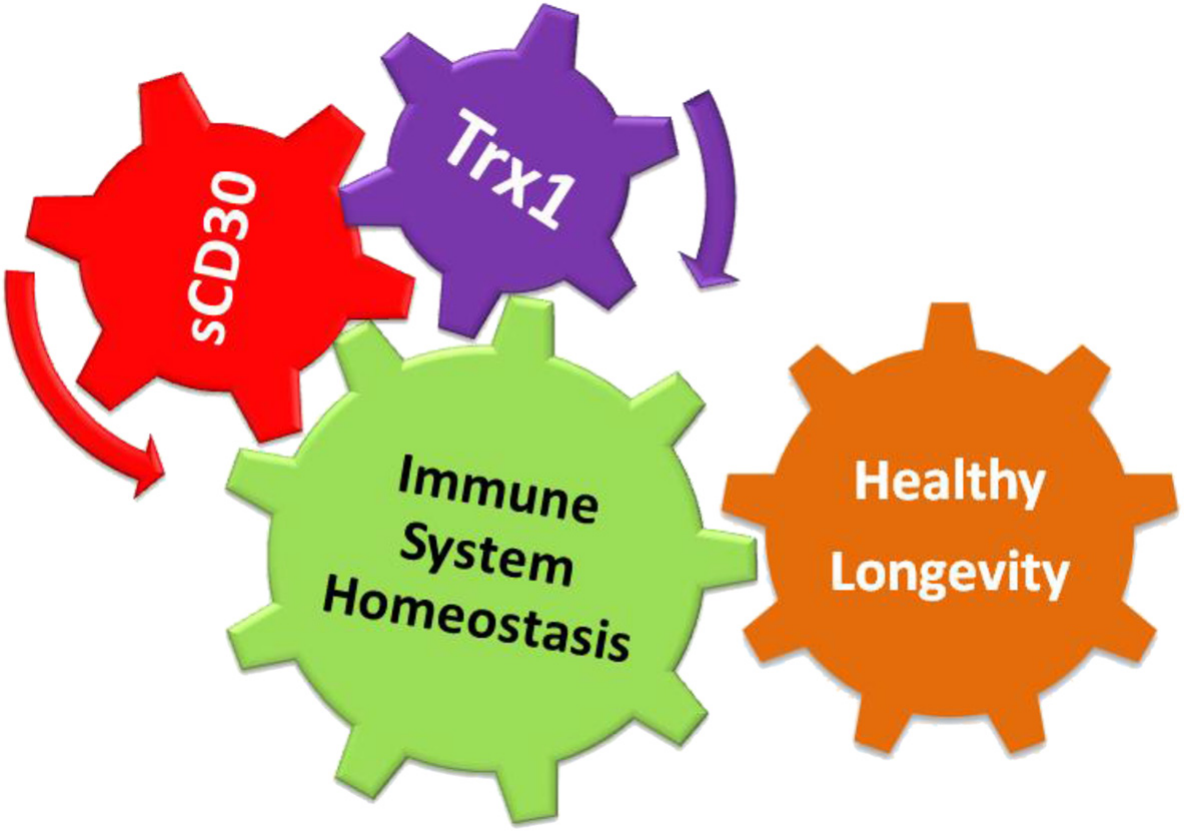
**The functional link between Trx1 and CD30 is a very important step in the physiologic homeostasis and it underlines the big potentiality of these elements as clinical diagnostic and therapeutic targets.** The results of the research explained that in addition to Trx1, sCD30 is also able to influence the CD30R capacity to mediate the activation of intracellular signals for immune system homeostasis and healthy longevity, thanks to the inhibition of the binding between CD30L and RCD30: Trx1 makes this function catalytically, modifying the stoichiometric structure of RCD30; sCD30 makes the same function binding and blocking the binding site of CD30L, with which it has a strong affinity. For these reasons they have both to be considered for the use of the RCD30 as immunological and therapeutic biomarkers, because Trx1 and sCD30 could both influence the capacity of CD30R to mediate the activation of intracellular signals.

The results [77,79-82] underlined the importance of its physiopathologic function, clarifying that the interaction between RCD30 and sCD30 (released when RCD30 interact whit its ligand CD30L), controls the physiologic homeostasis in the immune and in the neurologic system by regulating the functions of monocytes and dendritic cells, mature and immature, to direct the T-helper cell (Th) differentiation in the respective subtypes (Th1, Th2, Th3, Th9 and Th17). These results explain, then, that the functional link between Trx1 and sCD30 is a very important step in the physiologic homeostasis and it underlines the big potentiality of these elements as clinical diagnostic and therapeutic targets (Figure 11).

Indeed, similarly to Trx1, sCD30 is able to influence the RCD30 capacity to mediate the activation of intracellular signals, through the inhibition of the binding between CD30L and RCD30: Trx1 makes this function catalytically, modifying the stoichiometric structure of RCD30 [70]; sCD30 makes this function blocking the binding site of CD30L, with which it has a strong affinity [82]. During inflammatory situations RCD30 is strongly expressed on the immune cells and, as a consequence, there is an increase of the sCD30 levels that is released in the extracellular environment and of the inhibition of the RCD30 signals [82].

The results have, also, underlined that the sCD30 level variations in the cellular environment (serum, tissue or tumoural microenvironment) could be used as a biomarker of the immune system homeostasis and of the benefit or risk of therapeutic response [77,79-81,83,84]: the sCD30 level within the normal physiological ranges is index of the immune system homeostasis and of the therapeutic benefit, while a significant increase of the sCD30 level is, on the other hand, index of immunological deficit and of the therapeutic risk.

For these reasons Trx1 and sCD30 have both to be considered as homeostatic biomarkers. Research indicate that changes of the Trx1 and sCD30 levels are functional extracellular biomarkers of the new Trx1/CD30 target (Figure 11), while the Treg/Th1/Th9/Th17 cytokines levels are functional biomarkers of the intracellular pathways for the prognostic and diagnostic/therapeutic stratification of patients [77,85-87].

Further clarification of the regulation pathways by means of Trx1 and sCD30 molecules, could lead to non-invasive treatments for the reestablishment of the immunological homeostasis and therapeutic response benefit. The potential of Trx1 and CD30 as single targets and biomarkers has already been described in literature, but the innovative hypothesis is in the combined use of Trx1 and sCD30 as a double target/biomarker (Trx1—sCD30) [84]. The rational of this hypothesis is that the Trx1—sCD30 target/biomarker concretize the diagnostic and therapeutic possibility to intervene on the multiple pathways of the redox and the immune system, opening to new and important perspectives for the degenerative diseases related with the oxidative stress, as tumors [84] and ageing [56].

### Indication for an "Evaluation System"

**Figure 12:**
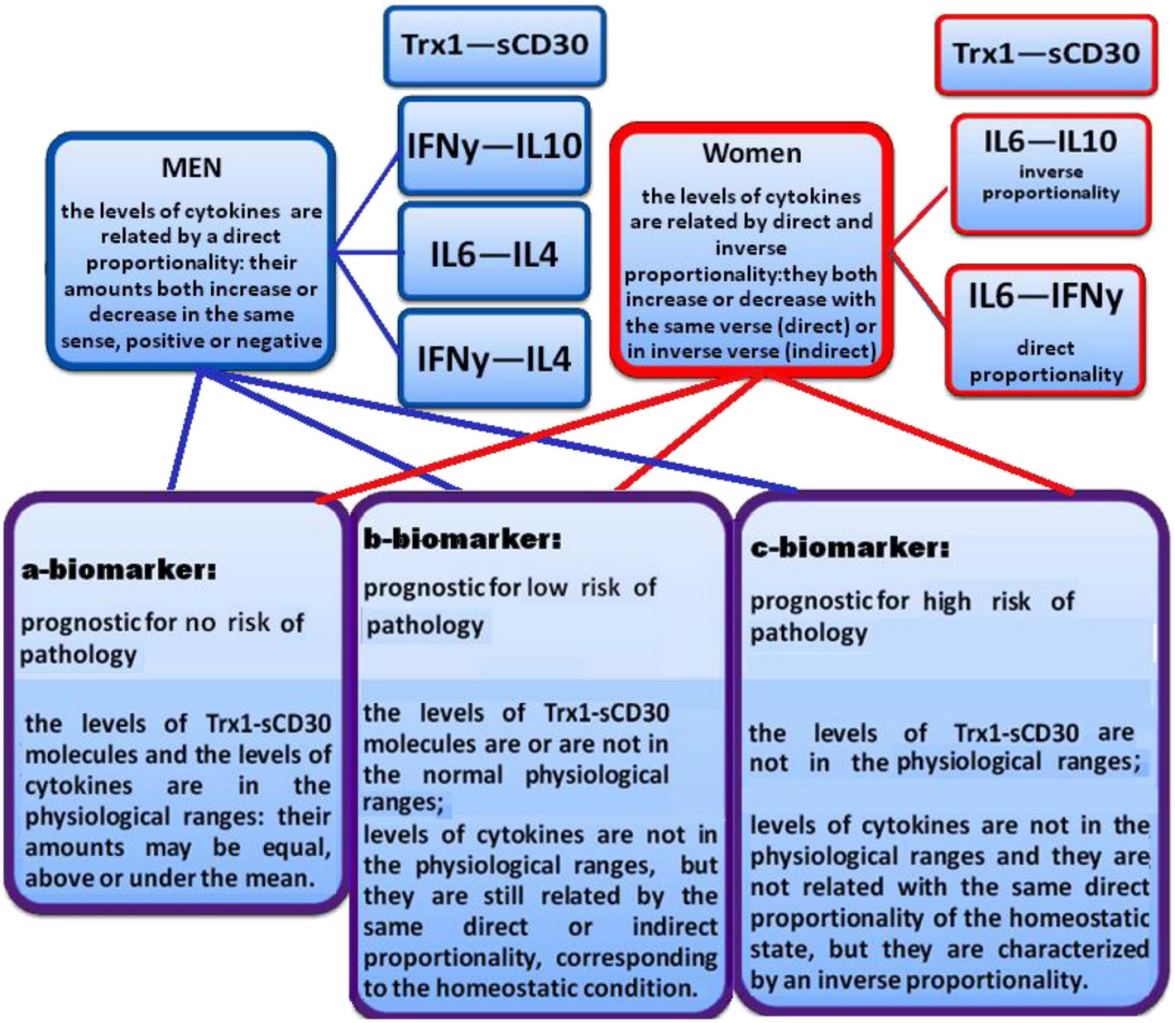
**Double biomarkers of homeostasis to evaluate the ability of our physiological system to control the inflammation and preserve the state of health: "Evaluation System".** The double biomarkers are of: 1) a type if the levels of Trx1—sCD30 molecules in both gender are within the normal physiological ranges and the levels of IFNy—IL10, IL6—IL4 and IFNy—IL4 for men are in the physiological ranges and they are related by a direct proportionality: their amounts may be equal, above or under the mean and they both increase or decrease in the same sense, positive or negative; the levels of IL6—IFNy and IL6—IL10 for women are in the physiological ranges, and they are respectively related by direct and inverse proportionality: their quantities are equal, above or under the mean, they both increase or decrease with the same verse, positive or negative, in case of direct proportionality, and in inverse verse, positive the first and negative the second or vice versa, in case of inverse proportionality; 2) b type if the levels of Trx1—sCD30 molecules in both gender are or are not in the normal physiological ranges and the levels of IFNy—IL10, IL6—IL4 and IFNy—L4 in men are not in the physiologic ranges, but they are still related by the same direct proportionality, corresponding to the homeostatic condition; the levels of IL6—IL10 and IL6—FNy in women are not in the physiological ranges, but they are still related by the same proportionalities, inverse and direct, corresponding to the homeostatic condition; 3) c type if levels of Trx1—sCD30 molecules in both gender are not in the normal physiological ranges and the levels of IFNy—IL10, IL6—IL4 and IFNy—IL4 in men are not in the physiological ranges and they are not related with the same direct proportionality of the homeostatic state, but they are characterized by an inverse proportionality; the levels of IL6—IFNy and IL6—IL10 in women are not in the physiological ranges and they are not related by the same inverse and direct proportionality of the homeostatic state, but the first couple by an inverse proportionality and the second by a direct one.

It consider different possibilities (Figure 12):

1. The levels of Trx1-sCD30 molecules and of gender-specific "cytokine couples" are within the normal physiological ranges: this condition is considered an a type biomarker, that is prognostic for the homeostatic state of the normal health condition. This is a biomarker of: a) subjects with no-risk of pathology in prevention strategies on healthy people; b) effective treatments, that are capable of restoring the physiological homeostasis in the control of inflammation, evidenced by the change of the patient's biomarker from the type "b" or "c" to the type "a".

2. The levels of Trx1-sCD30 molecules in both gender could be or not in the normal physiological ranges, but the level variations of gender "cytokine couples" (for example **IFNy—IL10, IL6—IL4** and **IFNy—IL4** in men) are out of the normal physiological ranges. In this particular case they change in a direction that agrees with the negative or positive trend (in this case positive) of their correlation in the normal healthy state, which is biomarker of homeostasis of b type. This is prognostic for the passage from the normal health condition of the homeostatic state, to transient inflammatory state. It indicates the restoration possibility of the homeostasis and it is an index of: a) subjects with low-risk of pathology in prevention strategies on healthy people; b) patient treatments that are not adequately effectives because they are capable of restoring the physiological homeostasis thanks to the control of the inflammation, but they have not yet restored the inflammatory base condition: this situation is evidenced by the no-change of the type "b" or "c" into the type "a" of the patient's biomarker.

3. The levels of Trx1-sCD30 molecules in both gender are not in the normal physiological ranges and variation of the levels of the cytokine couples (for example **IFNy—IL10, IL6—IL4** and **IFNy—IL4** in men) exit from the normal physiological rages, but in an opposite (in this case negative) direction in relationship with the normal health condition: this is considered a biomarker of the irreversible loss of homeostasis in the inflammation control, c type. This is prognostic for the passage from the transient inflammatory state and the restoration possibility of the control of inflammation, to the chronic inflammatory state and loss of physiological control of inflammation, in which its restoration is physiologically improbable. It is an index of: a) subjects with high-risk of pathology in prevention strategies on healthy people; b) patient treatments that are not effectives, because it is incapable of restoring the physiological homeostasis in the control of inflammation, that is evidenced by the no-change of the type "c" into the type "a" of the patient's biomarkers.

## Conclusions

The new research perspectives for the definition of “clinical biomarkers of homeostasis”, that could be prognostic for the risk of our physiologic system of losing the homeostatic capacity in the control of the inflammation, have a concrete and immediate relevance in the prevention of chronic-degenerative pathologies related with ageing. In fact, these biomarkers could lead to benefits in the improvement of a) the heath quality and lifespan for the elderly population, that is progressively increasing, and then b) the related budgets of the sanitary system administration.

The proposed clinical biomarkers (Figure 12) could be a real tool for the evaluation of the above homeostatic capacity, for the following reasons:

•The statistic methodologies of the system biology study have been used for their selection: they are analytic advanced methods used for the evaluation of the physiological network, amply consolidated and approved by the scientific community for the biomedical research [85-87]. This methodology, whose principal characteristic is to be suitable for the multiple component networks, comprehend the dynamic and functional evaluation of the relationships of all the variables (immune, genetic and clinical data) of the physiologic network in study. The analysis of all this amount of data is performed by “data driven, computational models”, that are mathematic models based on multiple experimental results: data are analyzed by factorial analysis and only the significant parameters are underlined.

•The "couple structuring" of the molecules, related with the proposed “homeostatic biomarkers”, is one of the determinant characteristics for the real prognostic capacity of these biomarkers. In fact the direct/indirect relationship between the levels of couples of molecules is a dynamic biomarker of the range of the homeostatic balance capacity of the physiologic system in the control of the inflammation. The same function could not be possible if the biomarker is constituted from only a molecule.

•The upper mentioned “double homeostatic biomarkers” are both, the same in both sexes (Trx1—sCD30) [84] and also gender-specific (cytokines couples) [52,55-58]. These are defined by couple of pro and anti-inflammatory cytokines of different type for men and women, that vary in an appropriate way controlling the homeostasis of the immune system and the inflammation in the respective genders: the sexual dimorphism of the immune response is one of the most relevant problems that have to be considered evaluating differences between men and women in the disease susceptibility and the therapeutic response.

•Finally, inflammatory and anti-inflammatory cytokines as well as sCD30 and Trx1 molecules are of basilar importance for the homeostasis control of the immune response and the inflammatory state: the interaction between the immune system and the redox one is very significant for this aim, because a key involvement of these two systems is concretely underlined by the relationship between the redox state, immune cell functioning and individual longevity.

For these reasons, “double homeostatic biomarkers” open to prevention programs for chronic-degenerative diseases on the healthy population that are not yet existent: they are suitable for the selection of healthy subjects with risk of developing this kind of pathologies. On these individuals is justified the application of preventing sanitary procedure, avoiding the application on other subjects when not motivated. They are also prognostic for the stratification of patients in clinic/therapeutic subgroups, allowing the quantification of the risk/benefit for the specific treatment, the selection of the most effective patient treatment and the development of a personalized medicine that will lead to positive transformation of the clinical strategies.

Further study are now required to verify the potential benefits in predictive medicine, that will validate or refute this new hypothesis.

## Competing interests

The authors of this manuscript have no conflict of interest to declare.

## Authors’ contributions

AMB designed the research, analyzed and interpreted data, wrote the manuscript. IC and PP contributed to design the research and analyze data; TDB contributed to analyze data; ES contributed to analyze data and in English writing of the text. GM, CDI, FG, FG, MB, GL, MV, MG, MF and MB contributed to design the research. All authors read and approved the final manuscript.
